# Prognosis value of IL-6, IL-8, and IL-1β in serum of patients with lung cancer: A fresh look at interleukins as a biomarker

**DOI:** 10.1016/j.heliyon.2022.e09953

**Published:** 2022-07-13

**Authors:** Xi Yan, Lina Han, Riyang Zhao, Sumaya Fatima, Lianmei Zhao, Feng Gao

**Affiliations:** a Department of Clinical Laboratory, The Fourth Hospital of Hebei Medical University, Shijiazhuang, 050011, China; b Research Center, The Fourth Hospital of Hebei Medical University, Shijiazhuang, 050011, China; c Department of Thoracic Surgery, The Fourth Hospital of Hebei Medical University, Shijiazhuang, 050011, China

**Keywords:** Lung cancer, IL-6, IL-8, IL-1β, Metastasis

## Abstract

Interleukins are assumed to be closely related to the occurrence and development of human malignant tumors, while a few of them were commonly used as diagnostic markers in clinical cancer, including lung cancer. This study aimed to explore the value of serum interleukin-1β (IL-1β), interleukin-6 (IL-6), and interleukin-8 (IL-8) combined with carcinoembryonic antigen (CEA) as biomarker panel for the diagnosis and metastasis prediction of lung cancer. IL-1β, IL-6, IL-8, and CEA in serum were determined using electrochemiluminescence immunoassay (ECLIA) and flow cytometry, and the diagnostic value of each marker was analyzed using receiver operating characteristic (ROC) curves and logistic fitting regression. We found that the levels of serum IL-1β, IL-6, and IL-8 showed no significant difference among squamous cell carcinoma, adenocarcinoma, and small cell carcinoma, while they were significantly higher in the lung cancer group or benign group than those in the healthy group. The levels of IL-8 and CEA were positively correlated with clinical stages respectively. Importantly, the panel of CEA + IL-6 + IL-8 has the highest efficacy for the diagnosis of lung cancer (AUC = 0.883) among all the detected panels, while the panel of IL-8 + CEA showed the most promising predictive value for the lymph node metastasis (AUC = 0.686) and distant metastasis of lung cancer (AUC = 0.793). In conclusion, IL-6 and IL-8 could be used as promising molecular biomarkers to diagnose and predict the metastasis of lung cancer independent of pathological types, improving the specificity and sensitivity of diagnosis for lung cancer when they were combined with CEA.

## 1 Introduction

Lung cancer has a high degree of malignancy, resulting in high morbidity and mortality worldwide [1]. According to data from the Global Cancer Observatory (https://gco.iarc.fr/) in 2018, 18.1 million new cases of cancer were found worldwide, among which lung cancer accounted for 18.4% of total cancer [2]. In China, 787000 new cases of lung cancer were diagnosed in 2015, with an incidence of 57.26/100000 and a mortality rate of 45.87/100000. At present, surgical treatment is still the first treatment for early and middle stage lung cancer [3], while due to the concealed symptoms of the early stage of lung cancer, most patients are already at an advanced stage when they firstly see a doctor. The latest data showed that the 5-year survival rate is only 18% when diagnosed in the late stage, while that can reach 73% when diagnosed in the early stage [4, 5]. Therefore, improving the efficiency of early diagnosis and accurately performing clinical staging of lung cancer is of great significance for improving the prognosis of lung cancer.

Inflammation is an important factor leading to a variety of cancers. Experimental data have shown that inflammation participates in the occurrence and development of almost all tumors, including in many ways [6, 7, 8, 9]. Interleukins (ILs) are important inflammatory cytokines. It is not only participated in tumorigenesis but also is expected to be a potential target for tumor therapy. IL-6 has been shown to exhibit increased production in various cancer types, including breast cancer [10], colorectal cancer [11], and lung cancer [12]. It has been demonstrated to exert several pro-malignant functions on cancer cells, such as the promotion of proliferation and metastasis [13]. IL-8 and IL-1β can induce angiogenesis in many ways and increase tumor invasiveness [14, 15, 16]. Accumulation studies have shown that the levels of IL-1 β, IL-6, and IL-8 are all associated with the occurrence and development of lung cancer [17, 18]. In brief, IL-1 β, IL-6 and IL-8 are potential to be used as diagnostic biomarkers in lung cancer. At present, research on ILs is mainly focused on their function of them in tumorigenesis and tumor immunotherapy [19, 20, 21]. In clinical application, the detection of ILs is mainly used to measure the body’s immune function or as a biomarker for infection [22, 23], not used as biomarkers for the diagnosis and metastasis prediction of lung cancer. Herein we hypothesized that the combination of ILs and carcinoembryonic antigen (CEA), one of the most used tumor markers [24], would improve the early diagnosis and prognostic evaluation of lung cancer.

This study retrospectively analyzed the level of serum CEA, IL-1β, IL-6, and IL-8 in patients with lung cancer, and the relationship between levels of these ILs with the pathological type and clinical stage of patients with lung cancer. We further evaluated the value of CEA, IL-1β, IL-6, and IL-8 for the auxiliary diagnosis and prognostic evaluation of lung cancer, to provide more accurate potential molecular biomarkers for the diagnosis of the patient with lung cancer.

## 2 Material and methods

### 2.1 Patients

Patients who were hospitalized in the Fourth Hospital of Hebei Medical University from March 2019 to July 2019 were selected as the subjects of this study. Altogether 133 patients with lung cancer including 24 Squamous cell carcinoma, 82 Adenocarcinoma, and 27 Small cell carcinoma, 81 males and 52 females, aged 37–83 years old, with a median age of 63 (56, 69); The benign group contained 24 patients, including 14 males and 10 females, aged 20–73 years old, with a median age of 61 (53, 67). The control group contained 72 healthy subjects, including 40 males and 32 females, aged from 24 to 75 years old, with a median age of 59 (56, 65). This study was approved by the Ethics Committee of the Fourth Hospital of Hebei Medical University (No. 2019057).

Inclusion criteria: (1) newly patients with lung cancer diagnosed by histopathology or cytology were selected into the lung cancer group; (2) patients diagnosed with the benign pulmonary disease by clinical examination were included in the benign group; and (3) healthy subjects composed the control group.

Exclusion criteria: (1) receipt of lung cancer-related drugs or surgical treatment; (2) incomplete case information; and (3) recent severe infection or use of immunosuppressant and other drugs.

### 2.2 Testing method

The peripheral blood was collected on an empty stomach in the early morning of the second day after diagnosis, and the serum was separated after centrifugation at 1000g centrifugal force for 10 min. Using multiple micro-spheres flow fluorescence immunoassay, using fluorescent microspheres as the solid phase of immune reaction, the levels of IL-8, IL-1 β, and IL-6 were detected by flow cytometry. The instruments and reagents used were Navios flow cytometry of BeckmanCoulter Company and the cytokine kit of Qingdao Raise Care Company of China. Using matched kits with a fully automated Cobas e602 immunoanalyzer (Roche Diagnostics GmbH, Germany), serum CEA level was detected according to the standard operation specification of electrochemiluminescence immunoassay (ECLIA). The experimental testing was carried out in strict accordance with standard operating procedures and the corresponding reagent instructions, the state of the testing instrument was stable, and the internal quality control was consistent. The following normal reference intervals were used: CEA: 0–5.0 ng/ml; IL-1β: 0–12.4 pg/ml; IL-6: 0–5.4 pg/ml; IL-8: 0–20.6 pg/ml. Comparative analysis of the levels of CEA, IL-1β, IL-6, and IL-8 among the three groups of subjects, different pathological types, and different clinical stages was performed. The clinical value of CEA alone and its combined detection with IL-1β, IL-6, and IL-8 for the diagnosis, lymph node metastasis, or distant metastasis of lung cancer was determined.

### 2.3 Statistical analysis

SPSS statistical 25.0 was used to analyze the data, and Prism 8.0 was used for auxiliary drawing. The metrological data of non-normal distribution are shown as the median (P_25_, P_75_). The Kruskal-Wallis test was used to study the comparison of each index among different groups and pathological types, and the levels among groups (I + II *vs*. III + IV) in different staging were compared by the Mann-Whitney test. Correlations were analyzed by the Spearman coefficient test. The prediction probability of each index was obtained by Logistic fitting regression, and then the ROC curve was analyzed to judge the value of the panel of biomarkers in the diagnosis and metastasis of lung cancer. An area under the ROC curve (AUC) less than 0.5 indicated poor diagnostic accuracy; while the closer the AUC was to 1, the higher the diagnostic accuracy. The difference was statistically significant (*p* < 0.05).

## 3 Results

### 3.1 Baseline data

There was no significant difference in age or sex among the three groups of patients (*p >* 0.05). The general characteristics of the subjects are listed in Table 1.

**Table 1:** Clinicopathological characteristics of the selected population.

Characteristics	Lung cancer group (n = 133)	Lung benign group (n = 24)	Control group (n = 72)	p- value
Age (years)	63 (56,69)	61 (53,67)	59 (56,65)	0.062
Gender				0.757
Male	81 (60.9%)	14 (58.3%)	40 (55.6%)	
Female	52 (39.1%)	10 (41.7%)	32 (44.4%)	
pathological types				-
Squamous cell carcinoma	24 (18.0%)	-	-	
Adenocarcinoma	82 (61.7%)	-	-	
Small cell carcinoma	27 (20.3%)	-	-	
Clinical stage				-
Stage Ⅰ/Ⅱ	63 (47.4%)	-	-	
Stage Ⅲ/Ⅳ	70 (52.6%)	-	-	
Lymph node metastasis				-
Yes	59 (44.4%)	-	-	
No	74 (55.6%)	-	-	
Distant metastasis				-
Yes	34 (25.6%)	-	-	
No	99 (74.4%)	-	-	

p-value, probability value.

### 3.2 Serum levels of CEA, IL-1β, IL-6, and IL-8 with regard to the pathological type

**Figure 1:**
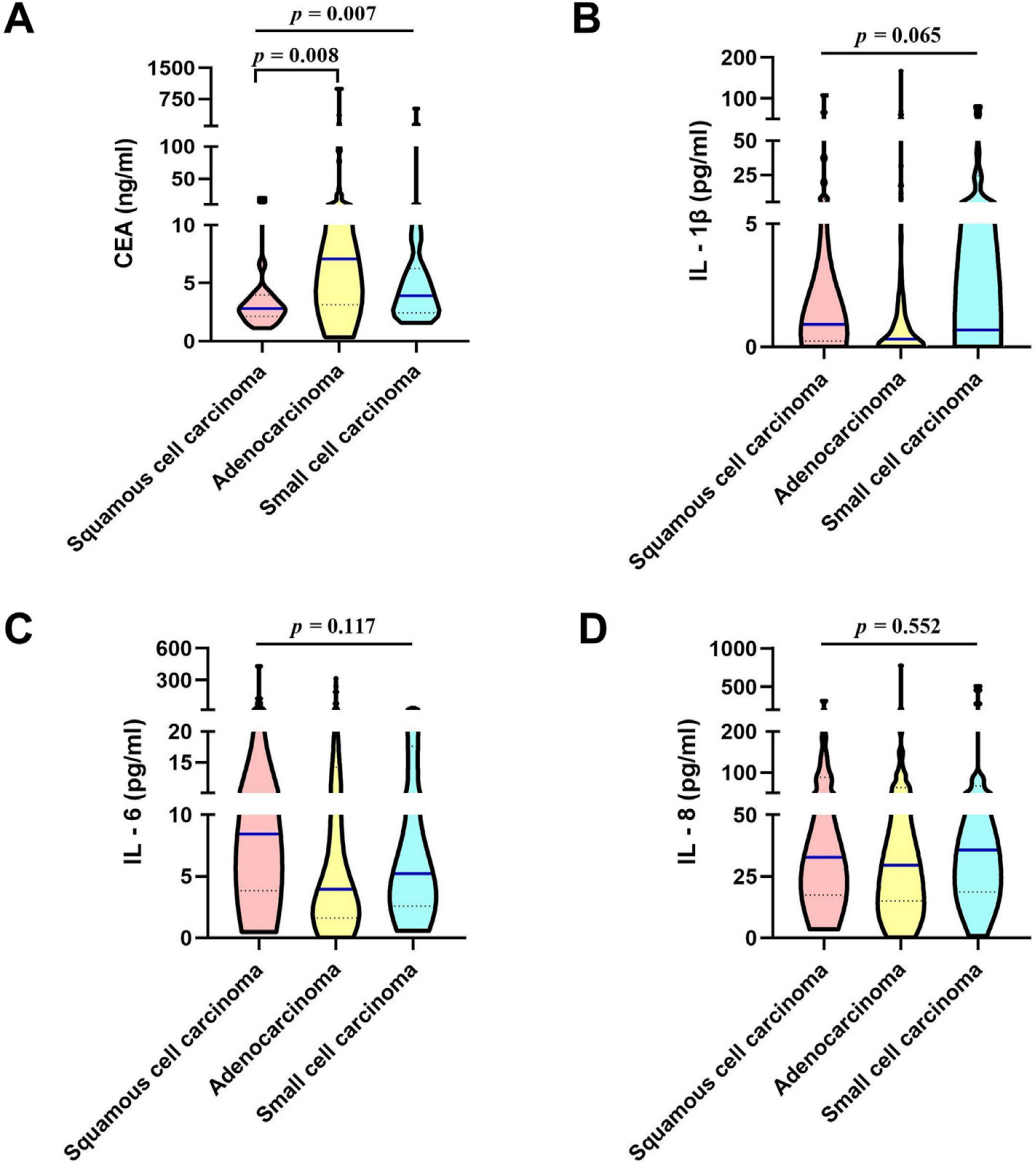
Distribution of serum CEA, IL-1β, IL-6 and IL-8 levels in patients with different pathological type: (A) CEA; (B) IL-1β; (C) IL-6; (D) IL-8.

To examine the association of IL-1β, IL-6, and IL-8 with pathological types, the Kruskal-Wallis test was first examined, as presented in Table 2 and Figure 1, the serum level of CEA in the adenocarcinoma group (6.74 ng/ml) was significantly higher than that in squamous cell carcinoma group (2.82 ng/ml) (*p* = 0.007), while the serum levels of IL-1β, IL-6 and IL-8 showed no significant difference among the three pathological types (*p* = 0.552, 0.065, 0.117). This allows us to analyze all lung cancer cases uniformly in the following studies without distinguishing pathological types.

**Table 2:** Serum levels of CEA, IL-8, IL-1β and IL-6 in patients with different pathological types [ M (P_25_, P_75_)].

Pathological types	n	CEA	IL-1β	IL-6	IL-8
Squamous cell carcinoma	24	2.82 (2.15, 3.98)	0.92 (0.24, 7.46)	8.44 (3.84, 29.25)	32.75 (17.40, 88.72)
Adenocarcinoma	82	6.74 (2.74, 15.58)	0.32 (0.00, 1.91)	3.95 (1.62, 14.23)	29.55 (15.05, 64.10)
Small cell carcinoma	27	3.92 (2.45, 6.26)	0.69 (0.06, 24.33)	5.22 (2.58, 17.63)	35.70 (18.70, 67.22)
H-value		9.823	5.477	4.289	1.189
p-value		0.007∗∗	0.065	0.117	0.552

∗∗p < 0.01. H*-*value, statistical value of the Kruskal-Wallis test; p*-*value, probability value; CEA, carcinoembryonic antigen; IL-1β, interleukin-1β; IL-6, interleukin-6; IL-8, interleukin-8.

### 3.3 Serum levels of CEA, IL-1β, IL-6, and IL-8 in the lung cancer group, benign group, and control group

**Figure 2:**
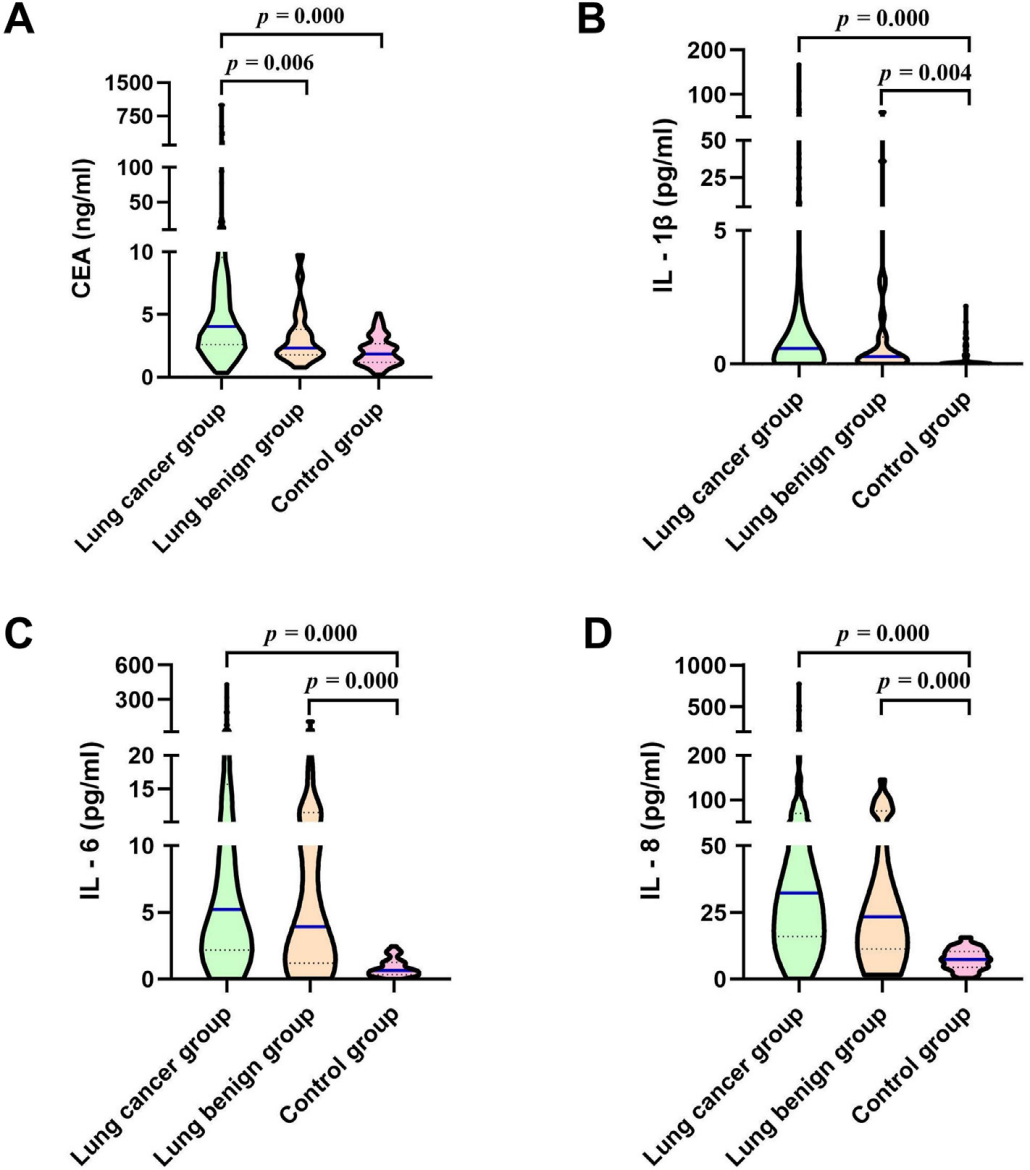
Distribution of serum CEA, IL-1β, IL-6, and IL-8 levels among the three groups: (A) CEA; (B) IL-1β; (C) IL-6; (D) IL-8.

Across the three groups, serum levels of CEA, IL-1β, IL-6, and IL-8 were highest in the lung cancer group, and the differences among the three groups were statistically significant (*p* = 0.000). The pairwise comparison shows serum levels of CEA, IL-1β, IL-6 and IL-8 in the lung cancer group were significantly higher than those in the control group (4.04 ng/ml *vs.* 1.86 ng/ml, 32.33 pg/ml *vs.* 23.43 pg/ml, 0.58 pg/ml *vs*. 0.28 pg/ml, and 5.22 pg/ml *vs.* 3.94 pg/ml) (*p* = 0.000), and the serum IL-1β, IL-6 and IL-8 levels in the benign group were higher than those in the control group (*p* < 0.01, Table 3 and Figure 2). The results suggest that serum IL-1β, IL-6, and IL-8 are highly expressed in lung cancer, which might be considered potential biomarkers for lung cancer.

**Table 3:** Comparison of CEA, IL-1β, IL-6 and IL-8 levels among the three groups [ M (P_25_, P_75_) ].

Groups	n	CEA	IL-1β	IL-6	IL-8
Lung cancer group	133	4.04 (2.61, 9.56)^a^^,^^b^	0.58 (0.00, 3.88)^a^	5.22 (2.20, 15.66)^a^	32.33 (15.99, 69.62)^a^
Lung benign group	24	2.33 (1.78, 3.82)	0.28 (0.05, 1.01)^a^	3.94 (1.20, 11.48)^a^	23.43 (11.34, 75.70)^a^
Control group	72	1.86 (1.18, 2.68)	0.00 (0.00, 0.21)	0.66 (0.35, 1.27)	7.40 (4.46, 10.46)
H-value		61.044	31.010	88.881	85.880
p-value		<0.001 ∗∗	<0.001 ∗∗	<0.001 ∗∗	<0.001 ∗∗

a p < 0.05, compared with the control group.

b p < 0.05, compared with the benign group; ∗∗ p < 0.01. H*-*value, statistical value of the Kruskal-Wallis test; p*-*value, probability value; CEA, carcinoembryonic antigen; IL-1β, interleukin-1β; IL-6, interleukin-6; IL-8, interleukin-8.

### 3.4 Relationships of the serum levels of CEA, IL-1β, IL-6, and IL-8 with clinical stage of lung cancer

**Figure 3:**
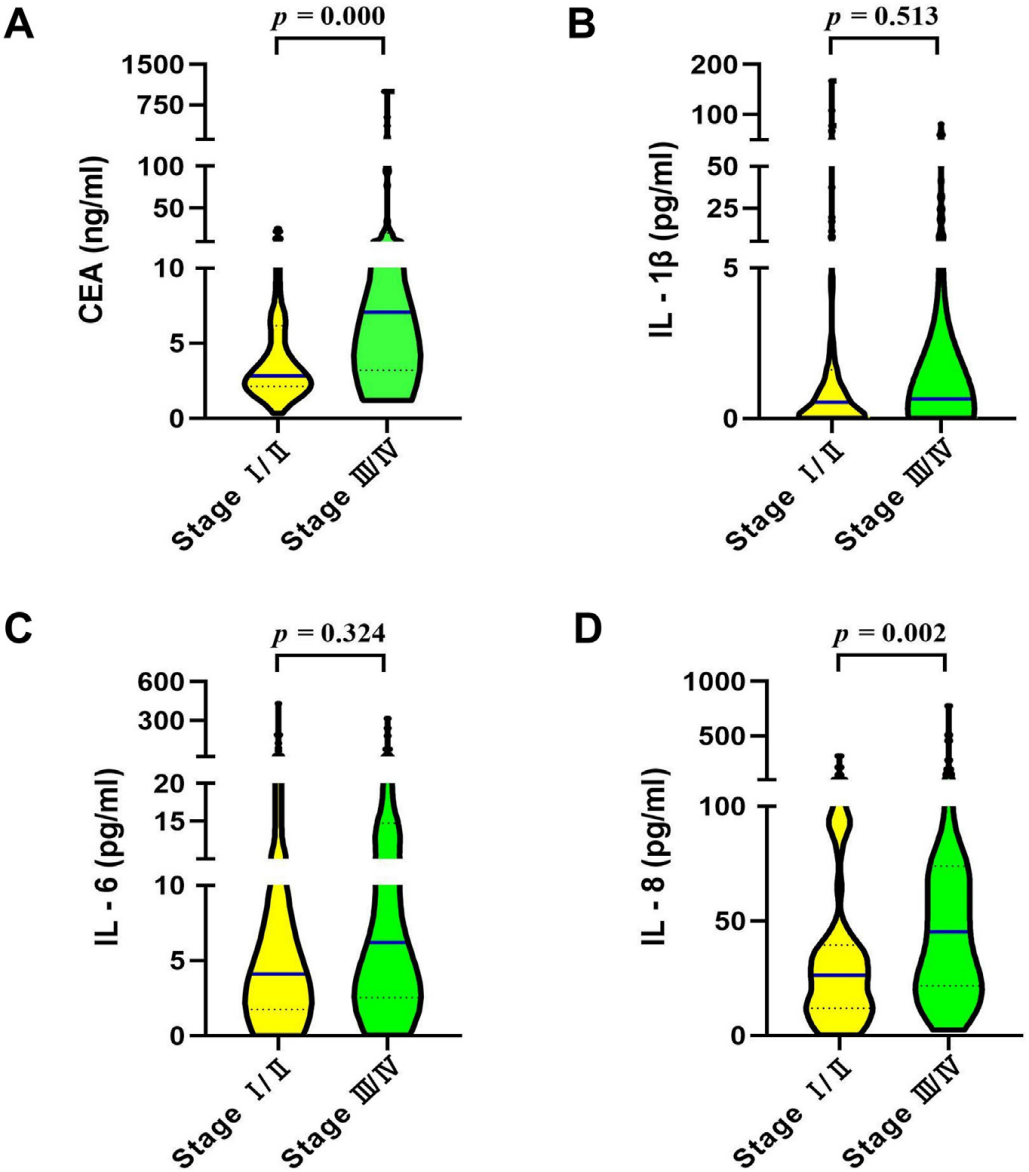
Distribution of serum CEA, IL-1β, IL-6, and IL-8 levels in patients with different clinical stage: (A) CEA; (B) IL-1β; (C) IL-6; (D) IL-8.

The association of CEA, IL-1β, IL-6, and IL-8 to clinical stages was examined; the level of serum CEA and IL-8 were associated with clinical staging. The levels of serum CEA and IL-8 in lung cancer patients at different stages are shown in Table 4 and Figure 3. Serum CEA and IL-8 showed higher expression in patients with stage Ⅲ/Ⅳ lung cancer (7.07 ng/ml, 45.21 pg/ml) than those in patients with stage Ⅰ/Ⅱ lung cancer (2.83 ng/ml, 26.37 pg/ml) (*p* = 0.000, 0.002), while there were no significant changes in the level of IL-1β or IL-6 between patients with stage Ⅰ/Ⅱ (0.54 pg/ml, 4.11 pg/ml) and stage Ⅲ/Ⅳ lung cancer (0.65 pg/ml, 6.21 pg/ml) (*p* = 0.511, 0.323). According to Spearman correlation analysis, the levels of serum CEA and IL-8 were positively correlated with the clinical stage (*p* = 0.000, 0.002), and the r-value were 0.394 and 0.264, respectively.

**Table 4:** Serum levels of CEA, IL-1β, IL-6 and IL-8 with regard to clinical stage [ M (P_25_, P_75_)].

Clinical Stages	n	CEA	IL-1β	IL-6	IL-8
Stage Ⅰ/Ⅱ	63	2.83 (2.12, 6.17)	0.54 (0.00, 1.62)	4.11 (1.75, 16.86)	26.37 (11.92, 39.49)
Stage Ⅲ/Ⅳ	70	7.07 (3.21, 20.07)	0.65 (0.00, 8.11)	6.21 (2.53, 14.74)	45.21 (21.73, 73.85)
Mann-Whitney test					
Z-value		-4.522	-0.657	-0.989	-3.033
P-value		<0.001∗∗	0.511	0.323	0.002∗∗
Spearman correlation analysis					
r-value		0.394	0.057	0.086	0.264
p-value		<0.001∗∗	0.513	0.324	0.002∗∗

∗∗p < 0.01. Z*-*value, the statistical value of the Mann-Whitney test; p*-*value, probability value; r-value, Spearman correlation coefficient; CEA, carcinoembryonic antigen; IL-1β, interleukin-1β; IL-6, interleukin-6; IL-8, interleukin-8.

### 3.5 Diagnostic efficacy of CEA, IL-1β, IL-6, and IL-8 expression in lung cancer

**Figure 4:**
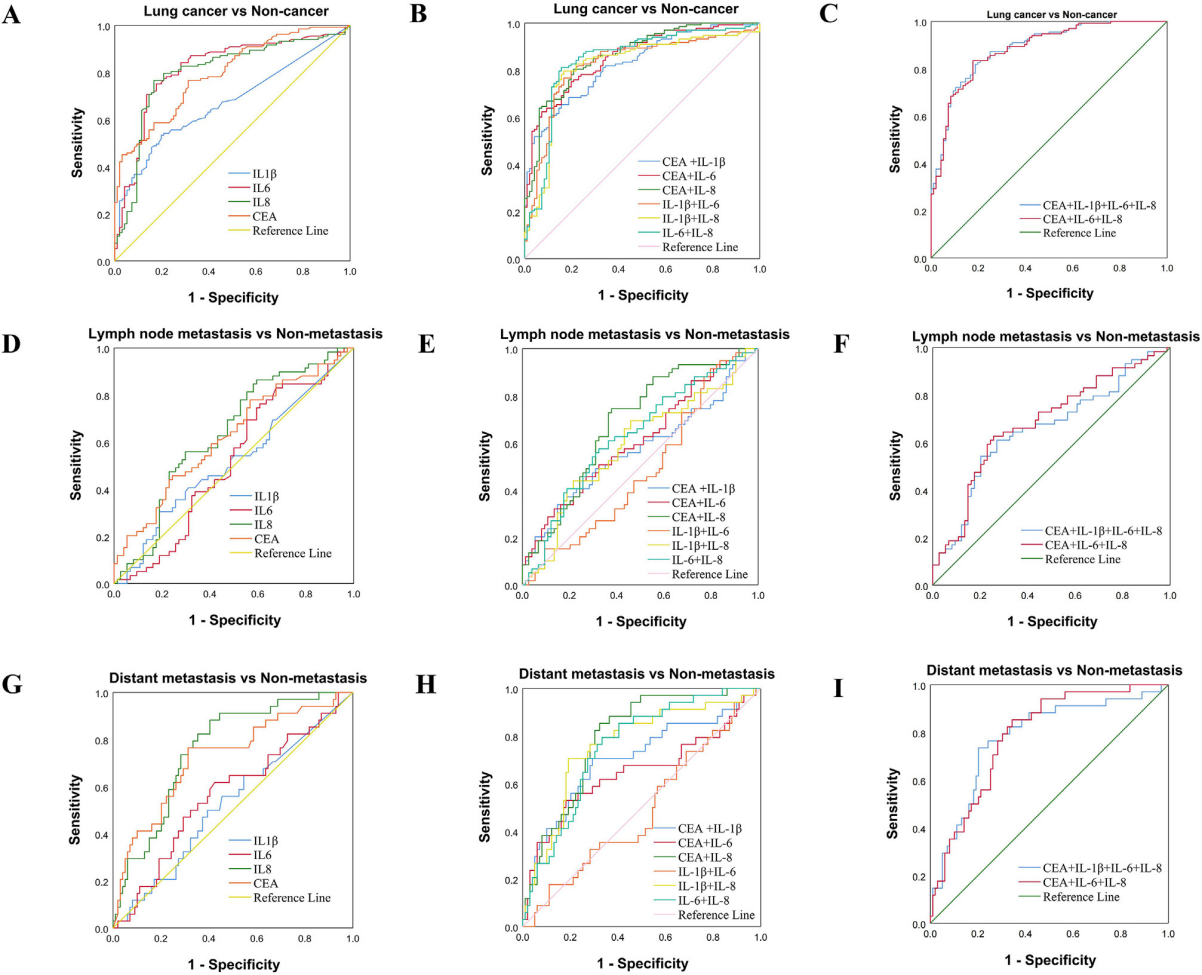
ROC curves of CEA, IL-1β, IL-6, IL-8 and the combination of lung cancer. (A–C) ROC curves for the diagnosis of lung cancer. (D–F) ROC curves for prediction of the lymph node metastasis of lung cancer. (G–I) ROC curves for the prediction of the distant metastasis of lung cancer.

To further confirm the diagnostic performance of IL-6, IL-8, and IL-1β as biomarkers of lung cancer, logistic fitting regression analysis was performed, and the ROC curve was drawn on the biomarkers individually and in combinations. As shown in Table 5 and Figure 4(A, B, C), the AUC value of IL-6 (0.817) was higher than the other three biomarkers, including IL-1β (0.671), IL-8 (0.800), and CEA (0.794). Likewise, IL-6 had the highest value with a 95 % confidence interval (0.758–0.875), and the sensitivity and specificity were 75.2% and 82.3%. Meanwhile, IL-8 shows the most Youden index (60.0), even if its AUC (0.800) is not the largest, indicating that it has a more preferable diagnostic effect as a diagnostic marker of lung cancer, with a sensitivity and specificity of 76.7% and 83.3%. Additionally, we further discovered that combinations of biomarkers lead to stronger diagnostic performance as compared to individual biomarkers. The panel of CEA + IL-1β+ IL-6 + IL-8 resulted in the highest AUC value (0.887) among all individual biomarkers and combinations. Interestingly, although the AUC of CEA + IL-6 + IL-8 was slightly lower (0.883), Youden’s index, sensitivity, and specificity were the highest, 65.8%, 83.5%, and 82.3%, respectively. These data indicated the combined analysis of multiple indexes will be more accurate than a single index, and the panel of CEA + IL-6 + IL-8 has the highest diagnostic value for lung cancer.

**Table 5:** Diagnosis Performance of Serum CEA, IL-1β, IL-6 and IL-8 for lung cancer.

Index	AUC	p-value	95% CI	Sensitivity (%)	Specificity (%)	Youden’s index (%)
IL-1β	0.671	<0.001∗∗	0.603–0.740	53.4	80.2	33.6
IL-6	0.817	<0.001∗∗	0.758–0.875	75.2	82.3	57.5
IL-8	0.800	<0.001∗∗	0.739–0.862	76.7	83.3	60.0
CEA	0.794	<0.001∗∗	0.737–0.850	76.7	68.7	45.4
IL-1β+IL-6	0.826	<0.001∗∗	0.769–0.883	79.7	80.2	59.9
IL-1β+IL-8	0.819	<0.001∗∗	0.759–0.879	78.2	84.4	62.6
IL-6+IL-8	0.844	<0.001∗∗	0.789–0.900	79.7	85.4	65.1
CEA + IL-1β	0.827	<0.001∗∗	0.775–0.878	65.4	84.4	49.8
CEA + IL-6	0.857	<0.001∗∗	0.810–0.905	75.2	80.2	55.4
CEA + IL-8	0.865	<0.001∗∗	0.818–0.911	80.5	79.2	59.7
CEA + IL-6+IL-8	0.883	<0.001∗∗	0.840–0.926	83.5	82.3	65.8
CEA + IL-1β+IL-6+IL-8	0.887	<0.001∗∗	0.845–0.929	82.0	81.2	63.2

∗∗p < 0.01. AUC, area under the curve; 95% CI, 95% confidence interval; p*-*value, probability value; CEA, carcinoembryonic antigen; IL-1β, interleukin-1β; IL-6, interleukin-6; IL-8, interleukin-8.

### 3.6 The predictive efficacy of serum CEA, IL-1β, IL-6, and IL-8 for lymph node metastasis and distant metastasis of lung cancer

We then evaluated the potential utility of the four biomarkers in lung cancer metastasis (Table 6 and Figure 4(D, E, F, G, H, I). Among the four single markers, only IL-8 and CEA have diagnostic significance in lymph node and distant metastasis of lung cancer (*p* < 0.05), and IL-8 was the most effective, with an AUC of 0.635 (95% CI: 0.540–0.730) for lymph node metastasis and 0.761 (95% CI: 0.674–0.848) for distant metastasis, which indicates that serum IL-8 expression exhibited good diagnostic performance in lung cancer metastasis.

**Table 6:** Predictive efficacy of Serum CEA, IL-1β, IL-6 and IL-8 for lymph node metastasis and distant metastasis of lung cancer.

Index	Lymph node metastasis					Distant metastasis				
	AUC (95% CI)	p-value	Sensitivity (%)	Specificity (%)	Youden’s index (%)	AUC (95% CI)	p-value	Sensitivity (%)	Specificity (%)	Youden’s index (%)
IL-1β	0.521 (0.421–0.621)	0.680	30.5	81.1	11.6	0.526 (0.414–0.638)	0.648	50.0	60.6	10.6
IL-6	0.516 (0.418–0.615)	0.746	76.3	39.2	15.5	0.569 (0.456–0.681)	0.231	61.8	57.6	19.4
IL-8	0.635 (0.540–0.730)	0.008∗∗	86.4	40.5	26.9	0.761 (0.674–0.848)	<0.001∗∗	88.2	59.6	47.8
CEA	0.624 (0.528–0.720)	0.014∗∗	45.8	75.7	21.5	0.726 (0.624–0.828)	<0.001∗∗	76.5	68.7	45.2
IL-1β+IL-6	0.484 (0.385–0.583)	0.751	91.5	20.3	11.8	0.488 (0.375–0.600)	0.833	17.6	88.9	6.5
IL-1β+IL-8	0.591 (0.493–0.690)	0.071	69.5	54.1	23.6	0.761 (0.665–0.857)	<0.001∗∗	70.6	80.8	51.4
IL-6+IL-8	0.632 (0.536–0.727)	0.009∗∗	57.6	67.6	25.2	0.755 (0.667–0.843)	<0.001∗∗	79.4	66.7	46.1
CEA + IL-1β	0.572 (0.471–0.673)	0.155	33.9	85.1	19.0	0.705 (0.595–0.815)	<0.001∗∗	70.6	70.7	41.3
CEA + IL-6	0.611 (0.514–0.707)	0.029∗	45.8	73.0	18.8	0.647 (0.525–0.769)	0.011∗	52.9	81.8	34.7
CEA + IL-8	0.686 (0.595–0.776)	<0.001∗∗	74.6	62.2	36.8	0.793 (0.713–0.873)	<0.001∗∗	85.3	67.7	53.0
CEA + IL-6+IL-8	0.682 (0.590–0.774)	<0.001∗∗	62.7	74.3	37.0	0.788 (0.707–0.869)	<0.001∗∗	85.3	65.7	51.0
CEA + IL-1β+IL-6+IL-8	0.655 (0.560–0.751)	0.002∗∗	61.0	73.0	34.0	0.780 (0.687–0.873)	<0.001∗∗	73.5	79.8	53.3

∗∗p < 0.01; ∗ p < 0.05. AUC, area under the curve; 95% CI, 95% confidence interval; p*-*value, probability value; CEA, carcinoembryonic antigen; IL-1β, interleukin-1β; IL-6, interleukin-6; IL-8, interleukin-8.

The predictive performance of IL-8 and CEA combined prediction models for predicting metastasis of lung cancer was also satisfactory, and the AUC value was higher than that of IL-8 or CEA alone. The panel of CEA + IL-8 had the best overall performance in predicting lymph node metastasis and distant metastasis of lung cancer, with AUC, sensitivity, and specificity of 0.686, 74.6%, and 62.2%, respectively, compared to 0.793, 85.3% and 67.7% for distant metastasis, and the remaining models showing overall lower performance. Interestingly, the combination of CEA and IL-8 resulted in low specificity but high sensitivity, however, the overall diagnostic performance was improved. Taken together, we should take into account both specificity and sensitivity when using the results of these comprehensive analyses.

## 4 Discussions

ILs are a class of cytokines with multiple effects produced by many cell types, and they are key factors regulating the immune response and inflammation. ILs play important roles in tumorigenesis, angiogenesis, tumor invasion and metastasis, and tumor microenvironment regulation by mediating multiple pathways. In the IL family, IL-1β, IL-6, and IL-8 are cytokines with a wide range of biological activities, which are involved in the occurrence and metastasis of many kinds of tumors, lung cancer is one of them [25, 26]. Tumor markers widely exist in the blood and bodily fluids, and their detection has the advantage of being simple and rapid, with samples easily obtained. At present, tumor markers have been widely used in the early diagnosis, treatment monitoring, and prognostic evaluation of tumors [27, 28]. CEA is a nonspecific tumor-associated antigen that is expressed in many types of tumor, but lacks specificity [29, 30].

A series of research has confirmed that the combined detection of inflammatory factors and tumor markers has a high value in the diagnosis of pancreatic cancer and colorectal cancer [31, 32]. In this study, we found that the levels of CEA, IL-1β, IL-6, and IL-8 in the lung cancer group were significantly higher than those in the healthy group, consistent with the reports in the literature [33, 34]. The serum level of CEA in the lung cancer group was higher than that in the benign lung disease group, but there were no significant differences in the level of IL-1β, IL-6, and IL-8 between the lung cancer group and the benign lung disease group. This may be due to the increases in cytokine levels caused by inflammation in other benign pulmonary diseases. The results of the analysis of these indexes and clinicopathological features showed that the level of CEA in serum of patients with lung adenocarcinoma was higher than that of lung squamous cell carcinoma and also higher than that of lung small cell cancer, which was consistent with a literature report [35], but there were no differences in IL-1β, IL-6 and IL-8 among different pathological types. Studies have shown that tumor markers have a high value in the clinical staging of lung cancer [36]. Our research showed that CEA and IL-8 are positively correlated with clinical staging. Compared with those patients with early-stage disease, serum levels of CEA and IL-8 in patients with late-stage lung cancer were significantly increased, but the levels of IL-1β and IL-6 had no change significantly, which is inconsistent with the results of Xie et al. [37] and may be related to the number of cases and the inclusion and exclusion criteria used.

In addition, we further analyzed the role of individual detection and combined detection in the auxiliary diagnosis of lung cancer, as well as the predictive value of lymph node metastasis and distant metastasis of lung cancer. From the results, we found that even though the panel of CEA + IL-1β + IL-6 + IL-8 has the largest AUC, the overall performance of CEA + IL-6 + IL-8 is superior (Youden’s index, sensitivity, and specificity are 65.8%, 83.5%, and 82.3% respectively). We believe that the panel of CEA + IL-6+IL-8 could be the most effectively used in the diagnosis of lung cancer. In addition, we also found that compared with the detection of CEA alone, the combination of CEA and IL-8 could improve the AUC for the lymph node and distant metastasis of lung cancer. These results indicate that the combined detection approach has good application value in predicting distant metastasis of lung cancer, but the sensitivity and specificity should be taken into account at the same time.

As a simple and rapid noninvasive examination, the combination of inflammatory cytokines and tumor markers is helpful to judge the clinical stage, critical metastasis, and distant metastasis of lung cancer and has good clinical application value. However, our study has some limitations for the limited number of cases included in this study and the retrospective design, the data obtained could be influenced by confounding factors, and further prospective studies are needed to confirm this conclusion.

## 5 Conclusion

In summary, our study suggests that serum IL-1β, IL-6, and IL-8 might serve as potential markers for the diagnosis of lung cancer. The panel of CEA + IL-6+IL-8 has the best diagnostic efficacy for lung cancer than CEA alone or CEA combined with IL-1β, IL-6 and IL-8 respectively; In addition, we also demonstrated for the first time that the panel containing IL-8 and CEA could be the promising molecular biomarker panel to predict the metastasis of lung cancer.

## Declarations

### Author contribution statement

Xi Yan: Performed the experiments; Wrote the paper.

Lina Han; Sumaya Fatima: Contributed reagents, materials, analysis tools or data.

Riyang Zhao: Analyzed and interpreted the data.

Lianmei Zhao; Feng Gao: Conceived and designed the experiments.

### Funding statement

Lianmei Zhao was supported by the National Natural Science Foundation of China [81772550].

Dr. Xi Yan was supported by the Key Project of the Health and Family Planning Commission of Hebei Province [20170757].

### Data availability statement

Data will be made available on request.

### Declaration of interest’s statement

The authors declare no conflict of interest.

### Additional information

No additional information is available for this paper.
